# The Role of Water Homeostasis in Muscle Function and Frailty: A Review

**DOI:** 10.3390/nu11081857

**Published:** 2019-08-09

**Authors:** Isabel Lorenzo, Mateu Serra-Prat, Juan Carlos Yébenes

**Affiliations:** 1 Research Unit, Consorci Sanitari del Maresme, 08304 Mataró (Barcelona), Spain; 2 Centro de Investigación Biomédica en Red de Enfermedades Hepáticas y Digestivas (CIBERehd), Instituto de Salud Carlos III, 08036 Barcelona, Spain; 3 Intensive Care Unit, Consorci Sanitari del Maresme, 08304 Mataró (Barcelona), Spain

**Keywords:** total body water, intracellular water, dehydration, osmotic stress, ageing, anabolic resistance, muscle mass, muscle strength, functional capacity, frailty

## Abstract

Water, the main component of the body, is distributed in the extracellular and intracellular compartments. Water exchange between these compartments is mainly governed by osmotic pressure. Extracellular water osmolarity must remain within very narrow limits to be compatible with life. Older adults lose the thirst sensation and the ability to concentrate urine, and this favours increased extracellular osmolarity (hyperosmotic stress). This situation, in turn, leads to cell dehydration, which has severe consequences for the intracellular protein structure and function and, ultimately, results in cell damage. Moreover, the fact that water determines cell volume may act as a metabolic signal, with cell swelling acting as an anabolic signal and cell shrinkage acting as a catabolic signal. Ageing also leads to a progressive loss in muscle mass and strength. Muscle strength is the main determinant of functional capacity, and, in elderly people, depends more on muscle quality than on muscle quantity (or muscle mass). Intracellular water content in lean mass has been related to muscle strength, functional capacity, and frailty risk, and has been proposed as an indicator of muscle quality and cell hydration. This review aims to assess the role of hyperosmotic stress and cell dehydration on muscle function and frailty.

## 1. Introduction

**Figure 1:**
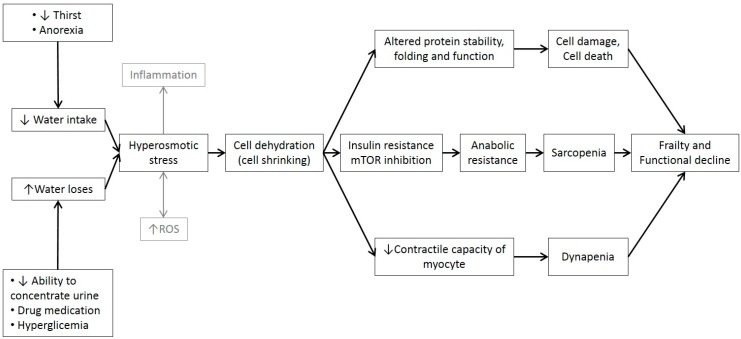
Summarizes the possible mechanisms by which water homeostasis alterations can affect muscle function and promote frailty. Reactive oxygen species (ROS).

Water is the main component of the body and represents approximately 76% of muscle mass. With ageing, there is a progressive decline in total body water (TBW) and intracellular water (ICW) parallel to an age-related loss of muscle mass and muscle strength. The elderly population is at an increased risk of low-grade chronic dehydration, which could presumably affect muscle function and the individual’s functional capacity. Loss of ICW with age is partially explained by the loss of muscle mass but may also be due to a reduction in cell hydration. The fact that age-related muscle mass loss is responsible for only a small part of muscle weakening suggests that strength in older individuals is more related to muscle quality than to muscle quantity. ICW determines cell volume and is believed to affect its metabolism, as water affects protein structure and enzymatic activity. Thus, ICW content in lean mass has been proposed as an indicator of muscle quality and cell hydration and has been related with strength, functionality, and frailty. However, the role of water in muscular function in the aged population is poorly understood and evidence is both scarce and scattered. Hypothesizing that progressive age-related dehydration may be responsible for muscle function impairment and frailty, we review cell dehydration mechanisms and the potential consequences for muscle function in the aged population (Figure 1). 

## 2. Water Structure, Properties and Biological Functions

The water molecule, while very simple, has unique and exceptional physical and chemical characteristics that have allowed life [1,2]. The electrostatic attraction between the positive H charge of one water molecule and the negative O charge of another water molecule allows a chemical union called hydrogen bonding [3]. This union is strong and determines both the high boiling point of water (breaking these bonds requires a lot of energy) and the liquidity of water in the temperature range favourable to life [4]. Water dissolves a large number of polar (or hydrophilic) substances (e.g., proteins, vitamins, glucose, urea, ions and gases), but does not dissolve apolar (or hydrophobic) substances (e.g., lipids) [5]. Hydrophobic reactions lead to the formation of cell membranes, micelles, and liposomes, the folding of proteins in tertiary and quaternary three-dimensional structures, and the double-helical structure of DNA [1].

Water has the following main functions in the human body:

(1) A metabolic function. Water is the medium in which all biochemical metabolism reactions occur. Water acts as a solvent and as a reactive in different metabolic reactions, mediates the recognition of molecules, acts as a communication channel between the inside and outside of proteins and increases the mobility or flexibility of enzymes, facilitating the enzymatic attack necessary for reactions to occur [6,7]. Thus, for example, the fact that each gram of muscle glycogen is stored with 2.7 g of water allows glycogen to be easily attacked by hydrolytic enzymes that quickly release glucose, the fuel for exercising muscles [1,8]. Apart from facilitating the enzymatic function, water also allows nervous transmission of electric current [5].
(2) A transport function. Circulating blood is the transport system that enables substances (nutrients, hormones, oxygen, metabolites, etc) to be exchanged between different organs and systems in body, while blood filtration by the kidneys eliminates the waste products of metabolism through the urine [1,9].
(3) A temperature control function. Water maintains a constant body temperature regardless of the ambient temperature and metabolic activity for several reasons: it has a high capacity to store energy in hydrogen bonds in such a way as to cushion temperature changes, it has high thermal conductivity which ensures rapid distribution and transfer of heat to the skin, and it requires a great deal of energy to be evaporated. By absorbing heat, distributing it among the liquid compartments of the body, and removing it through the skin through the evaporation of sweat, water keeps the body temperature within a very narrow range.
(4) A structural function. Water bound to cytoplasmic proteins determines cell volume, which, in turn, influences physiological mechanisms such as cellular performance and the regulation of cell proliferation or apoptotic cell death [10,11,12]. Water also determines plasma volume and perfusion of tissues.
(5) A mechanical function. Water acts as a lubricant in the mouth (through saliva), eyes (through tears) and joints (through synovial fluid), protects and promotes mucous membrane cleansing, and prevents injuries and fractures by adding flexibility and elasticity to tissues [9].

## 3. Water in the Body (Body Composition)

Water is the main component of the human body. It represents approximately 60% and 55% of body weight in adult men and women, respectively [13], and around 75% in children. Water content in lean mass (or fat-free mass) is about 70–75%, and in fat tissue is about 10%. Lean mass decreases with age and, because they have a higher fat mass percentage, is lower in women and in obese people [14]. Within the human body, water is distributed in intracellular and extracellular compartments. ICW, which represents 60% of TBW, is the main determinant of cell volume. Extracellular water (ECW), which represents the other 40% of TBW, includes plasma, interstitial fluid, and other transcellular fluids such as cerebrospinal fluid, synovial fluid, and vitreous humour [6]. Water is the solvent in the human body in which different solutes such as ions, proteins and other molecules are dissolved. The concentrations of these solutes are different between the intracellular and extracellular spaces and should be maintained within a small range of variation. The exchange of water between the intracellular and extracellular spaces is governed by the osmotic pressure exercised by the solutes on both sides of the cell membrane [15]. The most important intracellular ions are K^+^, Mg^2+^ and the phosphates and the main extracellular ions are Na^+^ and Cl^−^; the anion HCO3^−^ is common to both but is more abundant in the extracellular compartment [9]. The volume of the extracellular compartment (plasma and interstitium) depends mainly on total body sodium and the accompanying anions (mainly chloride and bicarbonate), since these constitute 90–95% of the total of osmotically active particles in extracellular fluid [9,16].

## 4. Water Homeostasis

### 4.1. Water Inputs and Outputs

The maintenance of a good body hydration status depends on how water inputs and outputs in the body are controlled and regulated [17]. Water comes from three main sources: food, beverages and metabolism. Food contributes approximately 20% of total water inputs, while beverages provide 70–75% [13]. The contribution of endogenous or metabolic water represents approximately 5–10% of total water input and amounts to about 250–350 mL per day in sedentary people [6]. There is great variability in the water intake recommendations of different international organizations [18,19,20,21,22,23,24]. Additional research is needed to establish specific water intake needs according to age, sex, physical activity, environmental temperature and humidity, type of diet, medication, and co-morbidities. Of clinical interest is an algorithm that would take into account the above cited factors to calculate individual water requirements and intake recommendations. Regarding body water outputs or losses, these are produced mainly by urine but also by faecal, cutaneous and respiratory excretion. The renal solutes load (RSL) represents the amount of metabolic waste by-products that must be eliminated by the kidney, while the obligatory urine water volume is the minimum amount of water eliminated by the kidneys that is required to eliminate the RSL. Thus, the obligatory urine water volume depends on the RSL, but also on the renal capacity to concentrate urine [9]. Its value is obtained by dividing the RSL by the urinary concentration value. The RSL in adults ranges between 600 mOsm/day and 900 mOsm/day and maximum renal concentration capacity is approximately 1200 mOsm/L of water. Therefore, minimum urine production is approximately 500 mL/day if maximum concentration capacity is achieved. If the ability to concentrate urine is reduced, then the amount of urine should be increased to ensure elimination of waste products [9], but water intake should also be increased to avoid dehydration. Insensible water loss is related to respiration and thermoregulation through sweat. It is estimated that approximately a quarter of human daily water intake is water lost during respiration (exhaled water) [25,26]. In charge of thermal control is the preoptic nucleus of the anterior hypothalamus, which receives information from thermal receptors in the skin. When a person’s core temperature increases, the thermoregulatory centre activates fibres in the autonomic nervous system to increase heat loss through cutaneous vasodilation (convection) and increased sweating (evaporation) [27].

### 4.2. Water Balance Control Mechanisms

Osmolarity is defined as the total concentration of particles in body liquids and is expressed as the number of solute osmoles (the number of particles that are osmotically active) per litre of solution (Osm/L). The osmolarity of body fluids needs to be maintained within very narrow values. The mechanisms that correct small osmolarity variations and water homeostasis in the body—which act fundamentally by stimulating thirst and renal excretion—are initiated by the stimulation of osmoreceptors, baroreceptors or certain hormones. Changes of 1–2% in plasma osmolarity above 280 mOsm/L stimulate the osmoreceptors located both peripherally (in the portal vein region) and centrally (in the preoptic region of the hypothalamus), activating the thirst centre and stimulating the release of arginine vasopressin (AVP) (the antidiuretic hormone) in the posterior part of the pituitary gland [28]. The main function of AVP is to increase water reabsorption in the kidneys [29]. Decreases of 10% in blood pressure stimulate the baroreceptors located in the pulmonary and renal arteries, which, in turn, stimulate the release of AVP and rennin. The latter causes an increase in circulating angiotensin II, leading to vasoconstriction and increased blood pressure [30]. The sensitivity of the AVP secretory response to an increase in plasma osmolarity increases when the volume of circulating blood decreases [28]. Therefore, osmotic and non-osmotic stimuli interact in a coordinated manner in the activation of thirst and the secretion of AVP [31,32,33]. While there are two receptors for AVP, V1 and V2, renal action occurs through V2. AVP plasma concentration ranges from 0.5 pmol/L (maximum diuresis) to 3–4 pmol/L (maximum urine concentration) [34]. In addition to the osmoreceptors and baroreceptors that stimulate AVP, there are other brain mechanisms that also contribute to controlling water homeostasis, including hormonal signals with effects on angiotensin II, relaxin and auricular natriuretic peptide (ANP) [35].

## 5. Osmolarity and Tonicity

Osmolarity and tonicity are two intimately related concepts, which, however, have important differences. As mentioned above, osmolarity refers to the amount of solutes per unit volume of solvent (the concentration of solutes) and is expressed in Osm/L units. Tonicity, which does not have units, refers to the ability of a solution to modify cell volume. A cell immersed in a hypotonic fluid will gain volume (water will enter the cell) and a cell immersed in a hypertonic fluid will lose volume (water will leave the cell). These effects depend on the osmolarity of the solution and on the ability of the solutes to cross the cell membrane. Not all isosmotic solutions are isotonic, not all hyperosmotic solutions are hypertonic, but all hyposmotic solutions are always hypotonic. For example, 0.9% saline and 5% glucose serum are both isosmotic solutions since they have an osmolarity of 278 mOsmol/L; however, if we administer them intravenously, saline is isotonic, since NaCl does not cross the cell membrane, while the glucose serum is hypotonic, since glucose enters the cell, with the resulting flow of water into the cell by osmotic pressure. Therefore, any pure glucose solution will be hypotonic, whatever its concentration or osmolarity. Moreover, since water passes through the cell membrane more rapidly than solutes, a hyperosmotic (and hypotonic) glucose solution will cause an initial loss, immediately followed by a gain, in cell volume so that glucose can be transported into the cell [36,37].

## 6. Transmembrane Water Transport: Aquaporins

Water passes through the cell membrane by facilitated diffusion, i.e., channelled by a family of transmembrane proteins called aquaporins (AQPs) that do not require energy for the transport. The AQPs regulate water flows between extracellular and intracellular spaces and play a key role in controlling cell volume. AQPs are assembled in the endoplasmic reticulum [38] and their expression and translocation to the plasma membrane is regulated very precisely by different stimuli, such as hormones (AVP), neurotransmitters and amino acids, as well as by hypo-osmolarity and hyperosmolarity [39]. Thirteen different AQPs have been described, expressed in different organs or tissues of the body. Despite some discrepancies between authors, they are broadly classified into three families: (1) selective water AQPs (AQP 0, 1, 2, 4, 5, 6 and 8), (2) aquaglyceroporins (AQP 3, 7, 9 and 10), which enable permeabilization of water, glycerol and urea and of other metabolically important small solutes such as carbon dioxide, nitric oxide, ammonium and hydrogen peroxide [40], and (3) intracellular AQPs (AQP 11 and 12) [41]. The AQPs are expressed mainly in organs that are involved in the absorption and regulation of water, i.e., the kidneys, gastrointestinal tract and secretory glands, as well as in cells, like the erythrocytes, that undergo constant osmotic changes [42]. For instance, when AVP binds to specific receptors in the collecting tubules (V2), it induces AQP2 expression in the luminal membrane of the medullary collecting tubule. AQP-2 expression in the kidneys forms water-permeable channels through which water is reabsorbed into the renal interstitium, decreasing urinary flow and increasing urinary osmolarity. There is growing evidence pointing to a role for the AQPs in different diseases. A lack of expression in some AQPs has been related to renal, cardiovascular, and inflammatory diseases (such as optic neuromyelitis) [43] and to obesity, diabetes, infertility, and cancer [44]. In addition, it has recently been observed that some AQPs can facilitate the diffusion of hydrogen peroxide (H_2_O_2_) (which is why they are also called peroxiporins, mainly AQP8), [45,46], nitric acid (NO), and gases such as CO_2_, NO, and O_2_. Hydrogen peroxide, with great oxidative power, is one of the main reactive oxygen species (ROS) produced by aerobic metabolism. An excess of ROS in cells leads to different cellular alterations related to metabolic, cardiovascular and renal diseases, ageing and frailty [44]. The dysregulation of peroxiporins (AQP8) may lead to oxidative stress and cell death [40,44]. Studies in animals have observed the presence of AQP4 in the cytoplasmic membrane of muscle cells (sarcolemma) and the presence of AQP1 in the endothelial cells of intramuscular capillaries. Both these AQPs are believed to be responsible for the rapid transport of water induced by exercise [47]. A reduction in AQP4 expression has been related to various muscular dystrophies [48] and, as a hypothesis that needs to be confirmed, may play a role in age-related muscle wasting. The contractile cycle in muscles seems to be associated with water entry and exit from muscle cells [48]; water participates in the conformational change that occurs between the actin and myosin filaments, passing from the union state of these proteins (weak complex) to the free state where the contraction is generated (strong complex) [49,50].

## 7. Dehydration in the Elderly

The aged population runs a greater risk of dehydration. Prevalence of dehydration in the elderly has been estimated at 20–30% and is associated with greater disability, morbidity and mortality [24,51,52]. Alterations in the hydro-electrolytic balance may cause decreased muscle strength, gait instability, falls, fractures, respiratory infections, confusion, renal failure and increased medication toxicity and may increase the risk of death [53,54,55]. Despite several studies showing that the elderly are at an increased risk of dehydration-related health problems, there is little information on suitable liquid intake and hydration in the elderly [56]. There are no clear and objective clinical signs of early dehydration in the elderly, but plasma osmolarity can be considered the gold standard for a diagnosis of dehydration. One study conducted in a sample of 2681 people aged 70–90 years old (Third National Health and Nutrition Examination NHANES III) reported that 40% had mild hypertonicity and 30% had severe or high hypertonicity [57]. Dehydration has more severe consequences in the elderly compared to young people because mechanisms to maintain body homeostasis in the elderly are altered [58]. The causes of dehydration in the elderly are numerous but are mainly related to a reduced thirst sensation (and, hence, water intake) and a reduced ability to concentrate urine. In individuals aged 60–79 years old, in comparison with younger adults, maximum urine osmolality and solute absorption are 20% and 50% lower, respectively [59]. This decrease in the ability to concentrate urine may be due to reduced AVP levels, yet several studies have shown that AVP levels in elderly people with plasma hyperosmolarity are increased, which would indicate a loss of renal receptor sensitivity to AVP (AVP resistance). Also affecting control of thirst and urinary excretion are changes in the functioning of the renin–angiotensin–aldosterone system (which stimulates vasoconstriction and renal reabsorption of Na and water) and alterations in atrial natriuretic peptide secretion by atrial myocytes in the heart (with functions opposite to those of aldosterone) [55]. High atrial natriuretic peptide levels reduce the thirst sensation and plasma levels of renin and aldosterone [58]. It is also important to remember that certain medications widely prescribed to the elderly promote water loss (diuretics, corticosteroids and metformin) and modify the thirst sensation (angiotensin 2 receptor antagonists, dopamine agonists and selective serotonin receptor inhibitors) [60]. Finally, other possible causes of dehydration in the elderly are a reduction in TBW due to reduced lean mass, diabetes, insulin resistance or hyperglycaemia states that favour plasmatic hyperosmolarity, and heat waves, which affect the elderly with greater intensity [61]. All these circumstances result in a decrease in TBW and ICW and favour low-grade chronic dehydration in aged individuals.

## 8. Hyperosmotic Stress in the Elderly

As mentioned, extracellular osmolarity needs to remain stable at 285–295 mOsm/L of water, regardless of the intake of water and solutes. Fluids with osmolarities below and above those limits are considered hypoosmotic and hyperosmotic, respectively. The increase in extracellular osmolarity (hyperosmotic stress) has important harmful effects for cells and leads to intracellular dehydration (cell shrinkage), in turn affecting the structure and function of proteins and altering intracellular enzymatic activity regarding the nucleus (DNA synthesis and repair), the mitochondria and the cytoskeleton [62,63]. The level of cell damage is proportional to the level of osmotic imbalance, while accumulated damage leads to apoptosis or cell death [63]. Cells have developed several mechanisms to compensate for hyperosmotic stress and to restore osmotic balance such as: (1) activation of ion transporters (non-selective channels for cations), involving entry of Na+ to balance osmolarity in the cell, with potential, however, to cause severe ionic imbalances, (2) synthesis of osmolytes, i.e., small intracellular organic inert molecules that are osmotically active, (3) induction of AQP gene expression to facilitate water movement, (4) rearrangement of the cytoskeleton to maintain cell volume, (5) activation of antioxidant enzymes to counteract the increase in ROS and oxidative stress resulting from water homeostasis alterations, and (6) activation of autophagic degradation [64]. Nonetheless, bodily osmotic balance is mainly regulated by AVP, which acts at the renal level to ensure water resorption and urinary concentration. The decline in renal function with age impairs the capacity to regulate and maintain osmotic balance. Hyperosmotic stress has been associated with:

(1) An important inflammatory response. The increased synthesis and secretion of different cytokines (Tumor Necrosis Factor-α, Interleukin-1β, Interleukin-6, Interleukin-8, Interleukin-18) may contribute to the development of chronic inflammatory diseases such as arthritis, inflammatory bowel disease and liver fibrosis [65,66]. Recent evidence also suggests that chronic inflammation may play an important role in carcinogenesis [67] and ageing [68,69].
(2) An increase in ROS production and oxidative stress [63,64,70].
(3) Diabetes, insulin resistance, and metabolic disorders. It has been observed that high levels of fasting AVP is a risk factor for type 2 diabetes and is associated with metabolic syndrome components (obesity, insulin resistance and hypertension). The explanation lies in the effect of AVP on adrenocorticotropic hormone (ACTH) and cortisol release [71]. Some studies have shown that high water intake decreases plasma osmolarity and AVP plasma levels and leads to greater glycaemic control, weight loss and reduced cardiovascular risk [72].
(4) Cardiovascular diseases. It has been observed that a chronic AVP increase in the elderly may worsen some diseases, such as hypertension and heart failure, and may increase the risk of thromboembolic events [71,72].
(5) Kidney disorders. High AVP levels have been associated with an increased risk of chronic kidney disease in two population-based cohorts in Sweden [72], although mechanisms and causes are unknown. Dehydration also promotes renal lithiasis.
(6) An increased risk of mortality [73].

## 9. Cell Volume Role in Cell Functions

Cell volume is mainly determined by water content and water transport through the cell membrane. Cell water outflow increases the concentration or density of intracellular macromolecules, with important consequences in terms of mechanical alterations, including increased cell stiffness, alterations in protein folding, protein transport, chromatin condensation, and stem cell differentiation [74]. Excessively dense macromolecules can alter protein stability, structure and function within the cell. It is known that the three-dimensional shape of proteins determines their activity and biological properties, and it has been suggested that the structure of proteins lacking a rigid and well-defined tertiary structure may be highly sensitive to changes in cell volume [75]; this fact could explain why disordered proteins act as sensors and activators of the cell cycle. Some authors have suggested that cell volume acts as a metabolic signal that regulates cell function (the cell swelling theory) [76,77]. In vivo evidence indicates that cell swelling leads to anabolism, counteracts proteolysis and stimulates glycogen synthesis, while decreased cell volume (cell shrinkage) promotes catabolism and protein degradation [12,77]. Kelleret al. [78] have suggested that plasma hypoosmolality promotes lipolysis and counteracts proteolysis and glycogenolysis, while hyperosmolarity induces glycogenolysis. Water binds to glycogen and ensures good availability of nutrients, optimizes energy resource use and promotes anabolism. ICW depletion negatively affects the availability of nutrients and may produce an intracellular catabolic effect. Nonetheless, more studies are needed to confirm the effect of glycogen availability on anabolism and protein synthesis, especially in the aged population, since some authors have reported that low availability of muscle glycogen does not compromise anabolic signalling or protein synthesis after resistance exercise stimuli in young healthy men [79]. The ECW/ICW ratio, as an indicator of cell quality and cell hydration, is high in critical patients and, when >1, is associated with proteolysis and poorer evolution in patients with sepsis and trauma [80,81,82]. This would suggest that ICW plays an important role in the functioning of cells.

## 10. Water’s Role in Metabolic Muscle Function

Approximately 45% of body weight in the elderly is muscle (a smaller percentage than in young adults) and the fact that 76% of that muscle is water indicates that muscle is the main reservoir of water in the body. In addition, studies of dehydrated mice show that the muscle and skin are the first and main organs to lose water, thereby protecting other vital organs such as the liver and the brain [83]. Dehydration therefore mainly affects the muscle and water loss can have important effects on both the mechanical and metabolic functions of muscle. Muscle is responsible for most glucose metabolism and plays a major role in the development of insulin resistance and in the treatment of type 2 diabetes mellitus (one of the most effective treatments for type 2 diabetes is physical exercise). Insulin promotes glucose uptake, mainly by the liver and muscle cells, by stimulating the expression of GLUT4 glucose transporters. Within the myocyte, glucose may be used immediately, may be stored as glycogen or may be expelled into adipose tissue, depending on existing myocyte glycogen reserves. Each gram of glycogen stored in human muscle is associated with about 3 g of water [1]. It has been suggested that glycogen and water recovery in muscle after exercise is a coordinated process [8], although more studies are needed to confirm this finding. It has also been reported that cellular hydration is a critical factor in triggering the cellular metabolism stimulated by insulin [84]. While ICW loss seems to decrease the effect of insulin on adipocytes and hepatocytes, its effect on muscle cells have not been studied [85]. Dehydration associated with hyperosmotic stress inhibits the mammalian target of rapamycin (mTOR) pathway, which, in turn, favours insulin resistance and decreases cell glucose uptake and glycogen synthesis (Figure 1). This effect of intracellular dehydration on mTOR and insulin resistance may vary depending on the type of cell, suggesting that further studies are required for myocytes [85]. If this effect of ICW on muscle cells were confirmed, it would represent a huge advance in our understanding of the pathogenesis of sarcopenia, dynapenia and frailty, since insulin, along with other hormones such as insulin-like growth factor 1 (IGF-1), are a requirement for muscle growth, muscle regeneration and protein synthesis through the mTORC1 pathway [86]. Although studies in relation to the effect of age on IGF-1 and mTORC1 are somewhat contradictory [87,88,89,90], there is growing evidence to suggest that anabolic resistance, defined as the inability of anabolic stimuli to stimulate muscle protein synthesis, is the main determinant of age-related muscle loss [91,92]. As mentioned, this anabolic resistance may be related to intracellular dehydration, but more studies are required to confirm this hypothesis. Moreover, as previously mentioned, hyperosmotic stress and cell dehydration lead to intracellular accumulation of abnormal, aberrant, damaged, aggregated, misfolded, or oxidized proteins, which, in turn, activate autophagy and ubiquitin proteasome pathways, i.e., the main cellular protein degradation and protein turnover mechanisms [93]. Autophagy is a self-protective mechanism that degrades dysfunctional cellular organelles and proteins by fusing with lysosomes. It acts as a catabolic pathway that maintains homeostasis within cells exposed to stressors such as nutrient deprivation or dehydration. Autophagy may be mediated by AMPK-dependent mTOR inhibition [93,94].

## 11. Water’s Role in Mechanical Muscle Function

It has been observed that muscle dehydration affects muscle contractile capacity [84,95]. The molecular mechanisms by which ICW affects muscle contractile capacity are, however, poorly understood, although some studies have suggested that, on the surface of hydrophilic substances (such as proteins), water is ordered or structured in such a way that increases its density and/or viscosity up to six times. This has been called the fourth phase of water, or exclusion zone water (EZ-water) since solutes are excluded [96,97]. The binding force of the hydrophilic surface with water is a key factor in the formation of the viscous interphase. EZ-water (negatively charged) envelopes all macromolecules in the cell and appears to play a central role in most of its metabolic and mechanical processes [97]. The increased viscosity of the water surrounding proteins does not favour the muscle contraction process. Water molecules in the relaxed myofibrils are extensively linked by hydrogen bonds (with very few free OH); however, when the muscle contracts, a significant disunion of the hydrogen bonds of the water that surrounds contractile proteins [50] leaves more free water molecules (more free OH) in the form of bulk water. In other words, some of the water bound to the contractile proteins is released when the muscle is activated [49], due to the breakage of hydrogen bonds and the destructuring of EZ-water. Nevertheless, it is still not clear which of the two phenomena occurs first: the changes in protein conformation or the break in the hydrogen bridges that order the water [50]. Further studies are therefore needed to deepen our understanding of the role played by water in muscle contraction.

## 12. Cell Hydration, Functional Capacity and Frailty

As people age, they experience a progressive decline in muscle mass and muscle strength. The loss of muscle strength is the main determinant of the decline in functional capacity observed in the elderly, as well as the main determinant of disability and dependence [98,99,100]. While it was generally believed that loss of muscle mass was the main cause of loss of muscle strength, more recent studies indicate that loss of muscle mass is responsible for less than 10% of muscle strength loss [101,102]. The age-related decline in muscle strength is faster and more pronounced than the age-related decline in muscle mass, and many studies that have evaluated the effect of physical exercise programmes on the maintenance or improvement of muscle mass have not been able to demonstrate a parallel improvement in muscle strength [103]. That is why some authors propose restricting the term sarcopenia to the loss of muscle mass that appears with age and differentiating sarcopenia from dynapenia, which refers to the loss of muscular strength with age [101,104]. Muscular strength is determined by many factors and, in the elderly, it seems to be more related to the quality of the muscle and its ability to contract and generate strength than to the quantity of muscle. Few studies have evaluated the effect of intracellular hydration on muscle function in the elderly, but some authors have suggested that muscle intracellular hydration could be an indicator of muscle quality and contractile capacity [105,106,107,108]. Ritz reported an increase in ECW with ageing, especially among people with disabilities or altered health conditions [106]. Yamada et al. [107] showed that the ECW/ICW ratio was a predictor of knee extension force and gait speed, independent of age, sex, and muscle mass; this would suggest that the age-related expansion of the extracellular space (or contraction of the intracellular space) is an indicator of muscle quality and is responsible, at least partially, for a reduced muscle strength/muscle mass ratio. Other authors have corroborated these results; for instance, for a sample of community-dwelling elderly people aged 75 years and older, it was demonstrated that ICW (measured by bioelectrical impedance) was strongly related to muscle strength, functional capacity, gait speed and frailty, independently of age, sex, body mass index (BMI) and the number of co-morbidities [108]. While those results point to a protective effect of cellular hydration on weakness and functional decline, the possibility remains that the loss of ICW observed with age is due to the decrease in muscle mass, leaving it unclear whether the positive effect of more ICW is due to greater muscle quantity or to greater muscle quality (with better cell hydration). The same authors proposed a new indicator of muscle quality [109], expressed as ICW content per unit of lean mass (mL/kg), showing that this ratio decreases with age, is positively related to strength and functional capacity and is negatively related to frailty risk, regardless of age, sex and the number of co-morbidities. Those results point to a key role for intracellular hydration in muscle performance, functional capacity and frailty risk, yet should be understood as preliminary. Again, further studies are necessary to corroborate and confirm the role of cell hydration in the genesis of frailty.

## 13. Final Remarks

Water is an essential nutrient for life as it plays fundamental metabolic, transport, structural and temperature control roles in the body. Ageing is characterized by slow and progressive process of dehydration and hyperosmotic stress, which, a part from being related with inflammation, causes cell shrinkage and damage to intracellular protein structure and function (Figure 1). Several studies suggest that cell dehydration may have severe effects on muscle, leading to catabolism, anabolic resistance, and muscle wasting as well as impaired muscle contractile capacity. Some preliminary studies have shown that ICW content in lean mass is independently associated with muscle strength, functional capacity and frailty. The existing evidence reinforces the notion of a key role played by water in ageing and frailty processes; it also points to the importance of early detection and correction of water loss in the elderly (even sub-clinically). The above-mentioned effects of water on protein folding and function and on cell volume and metabolism would therefore suggest that more attention needs to be paid to the hydration status of elderly people. 

Frailty is a state of diminished functional reserves in different organs and systems, resulting in increased vulnerability to adverse health outcomes [110]. Frailty is associated with different deficits or co-morbidities [111,112] and with muscle wasting and poor muscle strength [113]. Some authors believe that those deficits are not unconnected but have a common and still unknown origin [113]. We presume to hypothesize that cellular dehydration may be associated with the origins of frailty. However, there are still many gaps and uncertainties about the role of water in frailty genesis, so further research is required to evaluate and deepen our understanding of certain key issues: age-related alterations in the ability to concentrate urine, the role of AQP expression in the muscle, the relationship between dehydration and anabolic resistance (for instance, comparing the effect of insulin or exercise on protein synthesis between well hydrated and dehydrated myocytes), and the relationship between hyperosmotic stress, inflammation, and ROS production. We suggest that there is a need to establish objective, valid, and reliable indicators of early dehydration in the aged population, in order to establish evidence-based personalized water intake recommendations for older adults and to assess the effectiveness of water treatments (with different electrolytic and other solute compositions) on muscle function, functional capacity, and frailty prevention.
